# Determinants of Genetic Structure in a Highly Heterogeneous Landscape in Southwest China

**DOI:** 10.3389/fpls.2022.779989

**Published:** 2022-04-25

**Authors:** Moses C. Wambulwa, Ya-Huang Luo, Guang-Fu Zhu, Richard Milne, Francis N. Wachira, Zeng-Yuan Wu, Hong Wang, Lian-Ming Gao, De-Zhu Li, Jie Liu

**Affiliations:** ^1^CAS Key Laboratory for Plant Diversity and Biogeography of East Asia, Kunming Institute of Botany, Chinese Academy of Sciences, Kunming, China; ^2^Germplasm Bank of Wild Species, Kunming Institute of Botany, Chinese Academy of Sciences, Kunming, China; ^3^Department of Life Sciences, School of Science and Computing, South Eastern Kenya University, Kitui, Kenya; ^4^University of the Chinese Academy of Sciences, Beijing, China; ^5^School of Biological Sciences, Institute of Molecular Plant Sciences, University of Edinburgh, Edinburgh, United Kingdom; ^6^Lijiang Forest Biodiversity National Observation and Research Station, Kunming Institute of Botany, Chinese Academy of Sciences, Lijiang, China

**Keywords:** longitudinal range gorge region, Southwest China, genetic diversity, climate, topography, anthropogenic factors, conservation

## Abstract

Intra-specific genetic diversity is a fundamental component of biodiversity, and is key to species adaptation and persistence. However, significant knowledge gaps still exist in our understanding of the patterns of genetic diversity and their key determinants. Most previous investigations mainly utilized single-species and/or a limited number of explanatory variables; so here we mapped the patterns of plastid genetic diversity within 15 plant species, and explored the key determinants shaping these patterns using a wide range of variables. Population-level cpDNA sequence data for 15 plant species from the Longitudinal Range Gorge Region (LRGR), southwest China, were retrieved from literature and used to estimate haplotype diversity (*H*_D_) and population pairwise genetic differentiation (*F*_ST_) indices. Genetic diversity and divergence landscape surfaces were then generated based on the *H*_D_ and *F*_ST_, respectively, to clarify the patterns of genetic structure in the region. Subsequently, we analyzed the relationships between plastid genetic diversity and 16 explanatory variables (classified as anthropogenic, climatic, and topographic). We found that the highest genetic diversity occurred in the Yulong Mountain region, with a significant proportion (~74.81%) of the high diversity land area being located outside of protected areas. The highest genetic divergence was observed approximately along the 25°N latitudinal line, with notable peaks in the western and eastern edges of the LRGR. Genetic diversity (*H*_D_) was weakly but significantly positively correlated with both Latitude (*lat*) and Annual Mean Wet Day Frequency (*wet*), yet significantly negatively correlated with all of Longitude (*long*), Annual Mean Cloud Cover Percent (*cld*), Annual Mean Anthropogenic Flux (*ahf*), and Human Footprint Index (*hfp*). A combination of climatic, topographic, and anthropogenic factors explained a significant proportion (78%) of genetic variation, with topographic factors (*lat* and *long*) being the best predictors. Our analysis identified areas of high genetic diversity (genetic diversity “hotspots”) and divergence in the region, and these should be prioritized for conservation. This study contributes to a better understanding of the features that shape the distribution of plastid genetic diversity in the LRGR and thus would inform conservation management efforts in this species-rich, but vulnerable region.

## Introduction

Intra-specific genetic variation is a key prerequisite for species persistence, since it provides the raw material for evolution to act upon (Frankham et al., 2010). Since this variation is critical for future adaptation to ecological upheavals, knowledge of the spatial patterns of genetic variation would be valuable in developing robust conservation guidelines across taxa and regions (Escudero et al., 2003), particularly in the context of global climate change. Research on the delimitation of the factors that determine within-population genetic variation across landscapes has therefore been intensified in the recent past (Fenderson et al., 2019; De Kort et al., 2021). It is generally accepted that patterns of genetic diversity in natural populations are strongly influenced by various ecological factors, including climatic, geological, and anthropogenic factors (Blanco-Pastor et al., 2019; Li et al., 2019; Yu et al., 2019). These factors influence the genetic structure of many plant and animal communities by promoting genetic bottlenecks, founder events, gene flow barriers, and by providing dispersal corridors (Ellegren and Galtier, 2016; Fan et al., 2017; Mairal et al., 2017).

The interplay of topography and climate is known to influence the patterns of genetic variation (e.g., Li et al., 2019). Geological processes create high-elevation mountains and deep river valleys (Badgley et al., 2017), whose interactions with climate factors ultimately affect the local patterns of intraspecific genetic variation. For instance, mountain ridges may serve as either barriers or dispersal corridors to allow for range shifts (Ohsawa and Ide, 2008; Tian et al., 2018), while along the elevation, mountains may increase the dissimilarity of microclimatic conditions along the elevational gradient. Such dissimilarity facilitates population differentiation through the interaction of both adaptive (natural selection) and non-adaptive (genetic drift and gene flow) evolutionary processes across environmental gradients (Chen et al., 2014; De Villemereuil et al., 2018). Additionally, the dramatic alteration of natural ecosystems by humans (FAO, 2018) for agriculture and infrastructural development (Liu et al., 2017; Yong et al., 2020) has led to habitat fragmentation, particularly for species inhabiting high-elevation areas (Feeley and Silman, 2010). Since landscape heterogeneity can influence gene flow and population connectivity, the rise in these anthropogenic land alterations may increasingly isolate certain populations, thus hinder species’ ability to remain in natural areas to persist and adapt in a rapidly changing environment (Di Marco et al., 2019). Despite the widely demonstrated effects of climatic, topographic, and anthropogenic factors on plant genetic structure (e.g., Gao et al., 2007; Liu et al., 2013; Blanco-Pastor et al., 2019; Li et al., 2019), no previous study has attempted to test the combined effect, or relative contributions, of these factors. Southwest China provides a perfect location for testing the combined effects of these factors on plant genetic structure, as the region is characterized by heterogeneous landscapes and a highly variable climate, as well as intensified anthropogenic activities.

The Longitudinal Range Gorge Region (LRGR) covers a total area of 410,251.64 km^2^ and is located in southwest China, mostly in Yunnan Province. The region supports a rich biodiversity, with high levels of species endemicity (Wu, 1987), and is therefore an important part of the biodiversity hotspot “Mountains of Southwest China” (Mittermeier et al., 2004), but only ~13.02% of its area has legal protection (Xu et al., 2017). Additionally, the LRGR is a renowned natural heritage area, with its northern edge hosting the Three Parallel Rivers region, which is a UNESCO World Heritage site in Yunnan Province, China (UNESCO, 2010). The Three Parallel Rivers region comprises three major rivers (Yangtze, Mekong, and Salween), generally oriented in the north-south direction, creating deep longitudinal gorges that are bounded by high glaciated peaks. This extraordinary geomorphological feature has been shown to drive genetic divergence in several plant species (e.g., Li et al., 2011; Liu et al., 2013; Meng et al., 2015). Based on such genetic differentiation within populations, Yu et al. (2019) identified areas with high genetic diversity in the north of LRGR, though their analysis was focused on the Tibetan Plateau, and only covered a small part of the LRGR. In addition, long-term stable climate refugia have been identified in the Yunnan-Guizhou Plateau and Sichuan Basin (Tang et al., 2018), areas that overlap with the LRGR. Such long-term stable refugia could harbor high levels of genetic diversity (Carnaval et al., 2009). Therefore, these studies underscore the urgent need to map out such areas and identify the factors that determine the observed patterns, hence allowing for more targeted and effective conservation strategies in the area. However, despite the high value accorded to “genetic diversity” as the most fundamental of the three levels of biodiversity (www.cbd.int/convention), its patterns and determinants remain unexplored in the LRGR.

Although the LRGR is associated with rich biodiversity and endemism, the ecosystem is known to be highly fragile (Pu et al., 2007), especially as a result of the significant spatio-temporal climatic variations in the region (Zheng et al., 2008; Fan et al., 2011; Shi et al., 2015; Duan et al., 2016; Cheng et al., 2020). Such ecosystem fragility has frequently been associated with mountainous areas, as they usually show relatively higher vulnerability to climate change, particularly warming (e.g., Thuiller et al., 2005; Lenoir et al., 2008). Other anthropogenic factors also pose a significant threat to ecosystem integrity and sustainability in Yunnan Province, China (Qiu et al., 2018; Shrestha et al., 2021). Indeed, the past few decades have seen an unprecedented escalation of human activities in the LRGR, particularly Yunnan Province; for instance, to stimulate the social and economic development in this region, the central and local governments have initiated infrastructural expansion projects (e.g., expansion of road and railway networks; Qiu et al., 2018 and references therein), and these initiatives could potentially disrupt a wide range of ecosystem services (Liu et al., 2006a,b; Liang et al., 2014).

The unique landforms of the LRGR, coupled with the intensified human activities in the region, provide an ideal opportunity to simultaneously test the relative effects of mountain-valley vicariance, the associated climatic variations, and anthropogenic influences on the genetic structure of plant communities. Although the influence of topographic and climatic factors on genetic diversity patterns in some parts of the LRGR (as well as its surroundings) has been explored to a considerable degree (Gao et al., 2007; Li et al., 2011; Liu et al., 2013; Luo et al., 2017; Fan et al., 2018; Yu et al., 2019), a holistic picture of the entire LRGR is lacking. For instance, the meta-analyses by Fan et al. (2018) and Yu et al. (2019) incorporated only a few topographic variables (longitude, latitude, and altitude) in their regression analyses and did not consider any climatic or anthropogenic variables, a fact that may have weakened the drawn conclusions. Furthermore, most previous studies (e.g., Gao et al., 2007; Li et al., 2011; Liu et al., 2013; Luo et al., 2017) were based on single species or taxa, limiting what can be inferred about the general spatial patterns of genetic variation. More generally, however, there has been no empirical determination of the role of anthropogenic effects in the region, and their interaction with climate and topography, in altering the distribution of genetic diversity. To bridge these knowledge gaps, we performed a meta-analysis on 15 plant species alongside a comprehensive dataset of 16 variables describing anthropogenic, climatic, or topographic factors. Though the chloroplast genome is generally more conserved and slow-evolving, hence generally limiting its use for assessing intra-population genetic diversity, our study exclusively utilized intergenic spacers, which have been shown to exhibit a relatively higher degree of variability (Raubeson et al., 2005). Against this background, we sought to determine the plant genetic structure in the LRGR and to test the hypothesis that the three categories of variables contribute unequally toward explaining the patterns of genetic structure in the LRGR. We aimed to (1) map the distribution of plastid genetic diversity and divergence, and (2) determine the relative contributions of climatic, topographical, and anthropogenic factors in shaping the plastid genetic structure of the LRGR. Our study will shed light on the interplay between geophysical and anthropogenic forces, and also guide policy decisions on biodiversity conservation in the region.

## Materials and Methods

### Study Area

**Figure 1:**
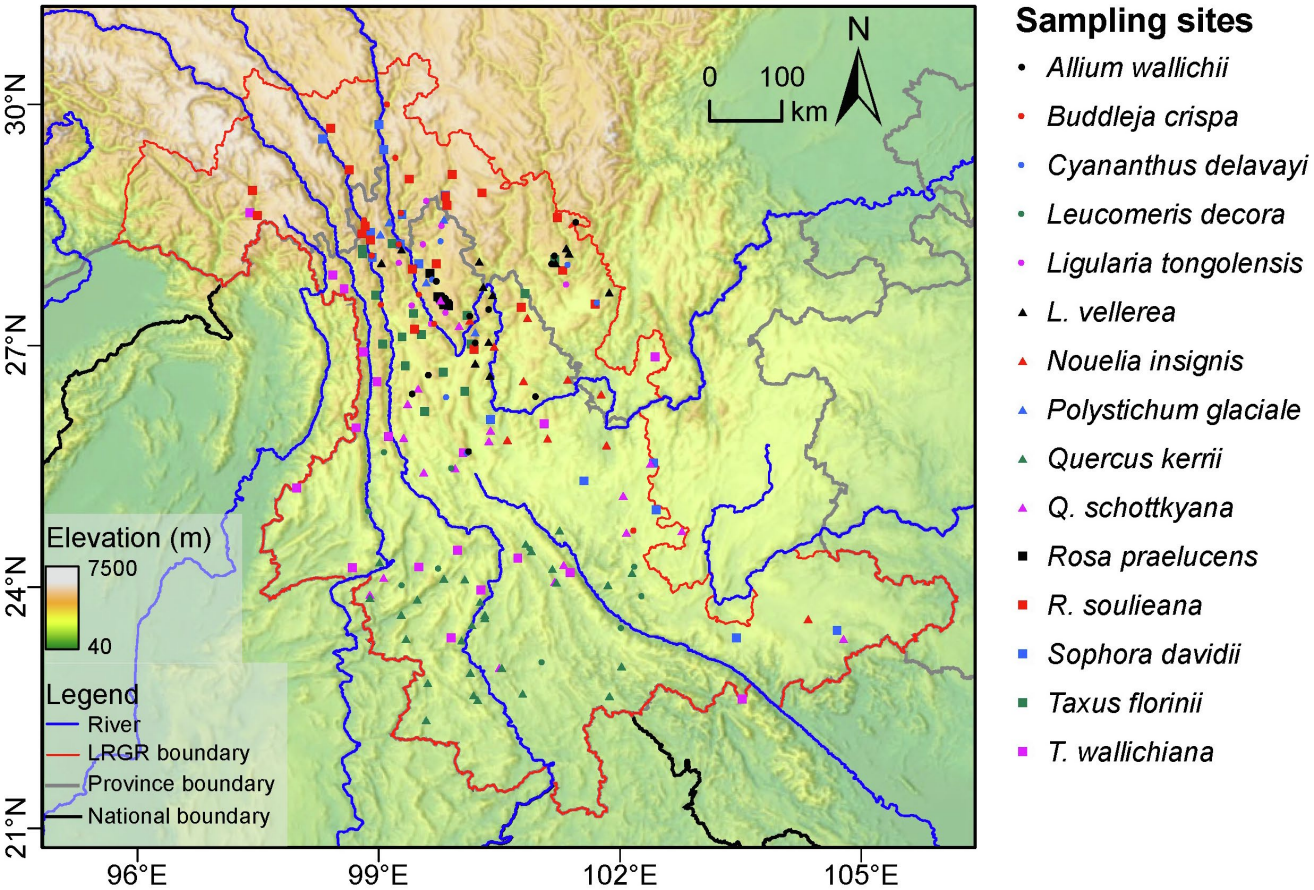
Map of the study area showing the distribution of the 15 species in the Longitudinal Range Gorge Region (LRGR).

The current study was restricted to the Longitudinal Range Gorge Region (LRGR). According to the boundary defined by Pan et al. (2012), the LRGR covers an area in southwest China that spans *ca*. 10.33 degrees of longitude (106.12–95.79°E) and *ca*. 9.45 degrees of latitude (30.60–21.15°N; Figure 1). Based on global 3 arc second (*ca*. 90 m) SRTM data (Farr et al., 2007), we estimated the average elevation of the LRGR to be 2369.06 m (above mean sea level), ranging from 76.4 to 6,740 m, and a total area of 410,251.64 km^2^. It spans both the subtropical and tropical monsoon climatic zones, with some parts of the high-elevation LRGR being temperate. Hence, temperatures vary widely with latitude and elevation, with the area supporting a diverse altitudinal zonation including tropical rain forests, subtropical evergreen broadleaf forests, and alpine vegetation.

### Collection of Genetic Data

**Figure 2:**
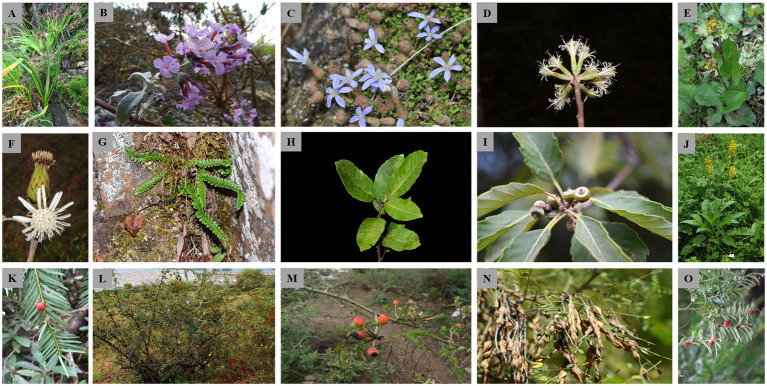
Representative individuals of the 15 plant species analyzed in the present study. **(A)** *Allium wallichii*; **(B)** *Buddleja crispa*; **(C)** *Cyananthus delavayi*; **(D)** *Leucomeris decora*; **(E)** *Ligularia tongolensis*; **(F)** *Nouelia insignis*; **(G)** *Polystichum glaciale*; **(H)** *Quercus kerrii*; **(I)** *Q. schottkyana*; **(J)** *Ligularia vellerea*; **(K)** *Taxus florinii*; **(L)** *Rosa praelucens*; **(M)** *R. soulieana*; **(N)** *Sophora davidii*; and **(O)** *T. wallichiana*. Permission to use the photographs was granted by the Germplasm Bank of Wild Species, Kunming Institute of Botany, Chinese Academy of Sciences.

We retrieved published articles on population genetics and phylogeography from Google Scholar.^1^ The literature search was conducted on 11/01/2019 using the terms “Hengduan Mountains” OR “southwest China” OR “Longitudinal Range Gorge Region” OR “Yunnan” OR “Three Parallel Rivers region” AND “plant” AND “phylogeography” AND “genetic structure” AND “cpDNA.” Among the retrieved articles, for consistency, we only considered studies that were based on chloroplast DNA (cpDNA) sequence markers; cpDNA sequences have been widely applied in population genetics and phylogeographic studies of plant species in the region; and hence, georeferenced population-level data are more easily accessible. Previously, cpDNA markers have been successfully applied to map genetic diversity patterns and clarify determinants thereof (Fan et al., 2018; Yu et al., 2019). Furthermore, studies in which the majority of the species range (>65%) fell outside the LRGR were excluded. Following these filtering steps, a total of 13 published studies, reporting genetic data for 15 plant species (Figures 1, 2; Table 1), met the selection criteria. For each of the populations of the 15 species, we gathered the geographic co-ordinate (longitude and latitude) from the articles and extracted their corresponding elevation data using the Extract Multivalues to Points toolbox in ArcGIS Pro v2.8.2.^2^ The referenced haplotype sequences were then downloaded from GenBank,^3^ using the accession numbers provided in the source studies.

**Table 1:** Details of the 15 plant species selected for analysis in the present study.

Species	Family	cpDNA marker(s)	Reference
*Allium wallichii*	Amaryllidaceae	*trnL-F*, *rps16*	Huang et al., 2014
*Buddleja crispa*	Buddlejaceae	*trnL-F*, *psbA-trnH*	Yue et al., 2012
*Cyananthus delavayi*	Campanulaceae	*trnH-psbA*, *psbD-trnT*	Li et al., 2012
*Leucomeris decora*	Asteraceae	*rpl32-trnL*	Zhao and Gong, 2015
*Ligularia tongolensis*	Asteraceae	*trnQ-5’rps16, trnL-rpl32*	Wang et al., 2011
*L. vellerea*	Asteraceae	*trnH-psbA and trnL-rpl32*	Yang et al., 2012
*Nouelia insignis*	Asteraceae	*rpl32-trnL*	Zhao and Gong, 2015
*Polystichum glaciale*	Dryopteridaceae	*atpB, rbcL*, *rpl32-trnL*, *trnL-F*	Luo et al., 2018
*Quercus kerrii*	Fagaceae	*psbA-trnH*, *ycf1*	Jiang et al., 2018
*Q. schottkyana*	Fagaceae	*psbA-trnH*, *trnT-trnL*, *atpI-atpH*	Jiang et al., 2016
*Rosa praelucens*	Rosaceae	*trnS-trnG*, *rpl20-rps12*, *trnS-trnfM*	Jian et al., 2018
*R. soulieana*	Rosaceae	*trnT-psbC*, *rpl20*-*rps12*	Jian et al., 2015
*Sophora davidii*	Fabaceae	*psbA-trnH*, *rpl*32-*trn*L	Fan et al., 2013
*Taxus florinii*	Taxaceae	*trnL-trnF*	Liu et al., 2013
*T. wallichiana*	Taxaceae	*trnL-trnF*	Liu et al., 2013

### Genetic Analyses

Based on the haplotype frequencies for each population and the mutation information in the haplotype sequences, we reconstructed the population and species sequence matrices in Geneious v2020.0.3^4^ using the MAFFT alignment algorithm (Katoh and Standley, 2013), and where necessary, alignments were trimmed to be of equal length. To ensure the validity of our population genetics analyses, only populations with a sample size of *N* ≥ 5 were retained for downstream analyses.

DnaSP v5.10 (Librado and Rozas, 2009) was used to define sequence sets, i.e., populations and to generate haplotype files for each of the 15 species. Subsequently, Arlequin v3.5.2 (Excoffier and Lischer, 2010) was used to calculate haplotype diversity *H*_D_ (Nei, 1987), nucleotide diversity *π* (Tajima, 1983; Nei, 1987), and population pairwise genetic differentiation *F*_ST_ (Tamura and Nei, 1993). As we detected a significant correlation (*r* = 0.728; *p* < 0.01) between *H*_D_ and *π*, we only used *H*_D_ in the subsequent analyses, since its calculation is insensitive to sample size, evolutionary forces, mode of reproduction, and ploidy level (Nei, 1973).

### Spatial Patterns of Plastid Genetic Diversity and Divergence

The population-level genetic diversity (*H*_D_) and pairwise divergence (*F*_ST_) values were used to generate genetic diversity and divergence landscapes using a modification of the Genetic Landscapes GIS Toolbox (Vandergast et al., 2011), which we implemented in ArcGIS Pro v2.8.2 with Python scripts. The landscape surfaces were generated at a resolution of 2.5 min (~5 km at the equator) based on the inverse distance weighting (IDW) interpolation method. Ordinarily, the interpolation method in the Genetic Landscapes Toolbox is based on the Minimum Bounding Geometry (convex hull type) for each species distribution point, which invariably leads to stretching of the boundary beyond the area where actual geometry exists. To resolve this challenge, we clipped the individual species landscapes to the boundary of the study region prior to combining them in the multi-species landscape, thus generating a multi-species landscape in which all the 15 species landscapes overlap throughout the study area. Subsequently, genetic diversity “hotspots” were defined as regions with standard deviations of genetic diversity >1.5 from the mean (Vandergast et al., 2008). Next, the geographic coverage of these hotspots was compared with that of protected areas, based on data obtained from the World Database on Protected Areas (WDPA),^5^ and validated with available information on China’s national nature reserves (You et al., 2018). To confirm whether the observed genetic differentiation was related to geographic distance, we performed isolation-by-distance (IBD) analysis for each species by testing correlation of the Euclidean geodistance with population pairwise genetic differentiation (*F*_ST_) using Mantel tests in the *vegan* package in R (Oksanen et al., 2011).

### Variables and Regression Analyses

A total of 66 explanatory variables (2 anthropogenic, 57 climatic, and 7 topographic variables) were extracted from various databases at various spatial resolutions (Supplementary Table S1). To avoid collinearity among the extracted variables within a given category, we omitted one variable for every pair that exhibited a high correlation coefficient (*r* > 0.7). After this, a total of 16 explanatory variables (10 climatic, 4 topographic, and 2 anthropogenic) were retained for subsequent analysis (Supplementary Table S2).

The response variable values (*H*_D_) were extracted from the extrapolated plastid genetic diversity landscape at the same spatial resolution as the corresponding explanatory variables (2.5 min resolution; Supplementary Table S2). To avoid bias that would be caused by the uneven number of variables across the categories, we selected only the two best variables in each of the climatic and topographic categories using the *importance* function within the Random Forest model implemented in the *randomForest* R package (Liaw and Wiener, 2002), plus the sole two in the anthropogenic category. Here, importance was defined as the degree to which the inclusion of each predictor decreases the residual model variance. We then carried out regression analyses on the selected variables with the corresponding *H*_D_ values based on generalized additive models (GAM) with integrated smoothness estimation using the “gam” function in the *mgcv* R package (Wood, 2017). Subsequently, the relative contributions of climatic, topographic, and anthropogenic factors, as well as their overlaps, in explaining the observed patterns of genetic diversity, were determined through redundancy analysis (RDA) and variation partitioning (Borcard, 1992) using the *vegan* R package.

## Results

### Plastid Genetic Diversity and Divergence Patterns

**Figure 3:**
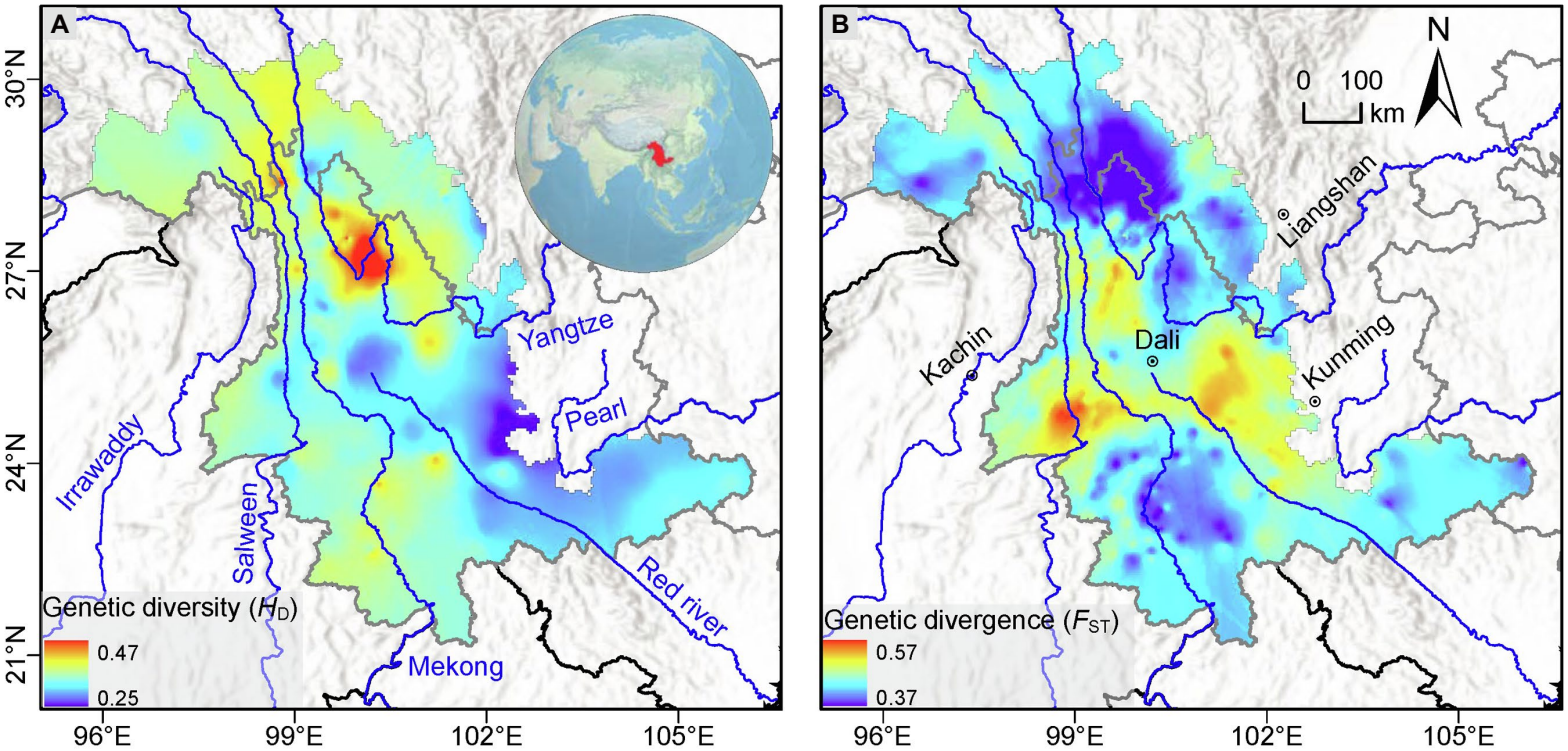
Multi-species genetic landscapes for **(A)** genetic diversity based on haplotype diversity (*H*_D_) and **(B)** population pairwise genetic divergence (*F*_ST_). The major rivers in the region are represented by blue lines.

Population-level genetic diversity indices varied widely, with nearly half (49.7%) of the populations lacking genetic variation (i.e., comprising a single detected haplotype), and ~5% showing maximum haplotype diversity (*H*_D_ = 1, meaning every sampled individual had a different haplotype; Supplementary Table S2). After interpolation on the LRGR landscape surface, the average genetic diversity across the 15 species ranged from 0.25 to 0.47 (Figure 3A). Our results indicated that the northern area of the LRGR generally harbors higher levels of genetic diversity, with the maximum haplotype diversity residing in the Yulong Mountain region, near the first major meandering of the Yangtze River. Relatively lower genetic diversity was observed toward the eastern edges of the study region (Figure 3A).

The interpolated genetic divergence (*F*_ST_) ranged from 0.39 to 0.59 and showed high divergence approximately along the 25°N latitudinal line, with notable peaks at the western and eastern ends of this line (Figure 3B). Conversely, relatively low genetic divergence between populations was observed for populations in the northern and southern peripheries of the study area. Based on IBD analysis, only 5 of the 15 species showed significantly positive correlation between geographic and genetic distances, i.e., *Leucomeris decora* (*r* = 0.3, *p* = 0.04), *Ligularia vellerea* (*r* = 0.47, *p* = 0.002), *Quercus kerrii* (*r* = 0.19, *p* = 0.01), *Rosa soulieana* (*r* = 0.39, *p* = 0.03), and *Sophora davidii* (*r* = 0.65, *p* = 0.001; Supplementary Table S3).

### Plastid Genetic Diversity Hotspots

**Figure 4:**
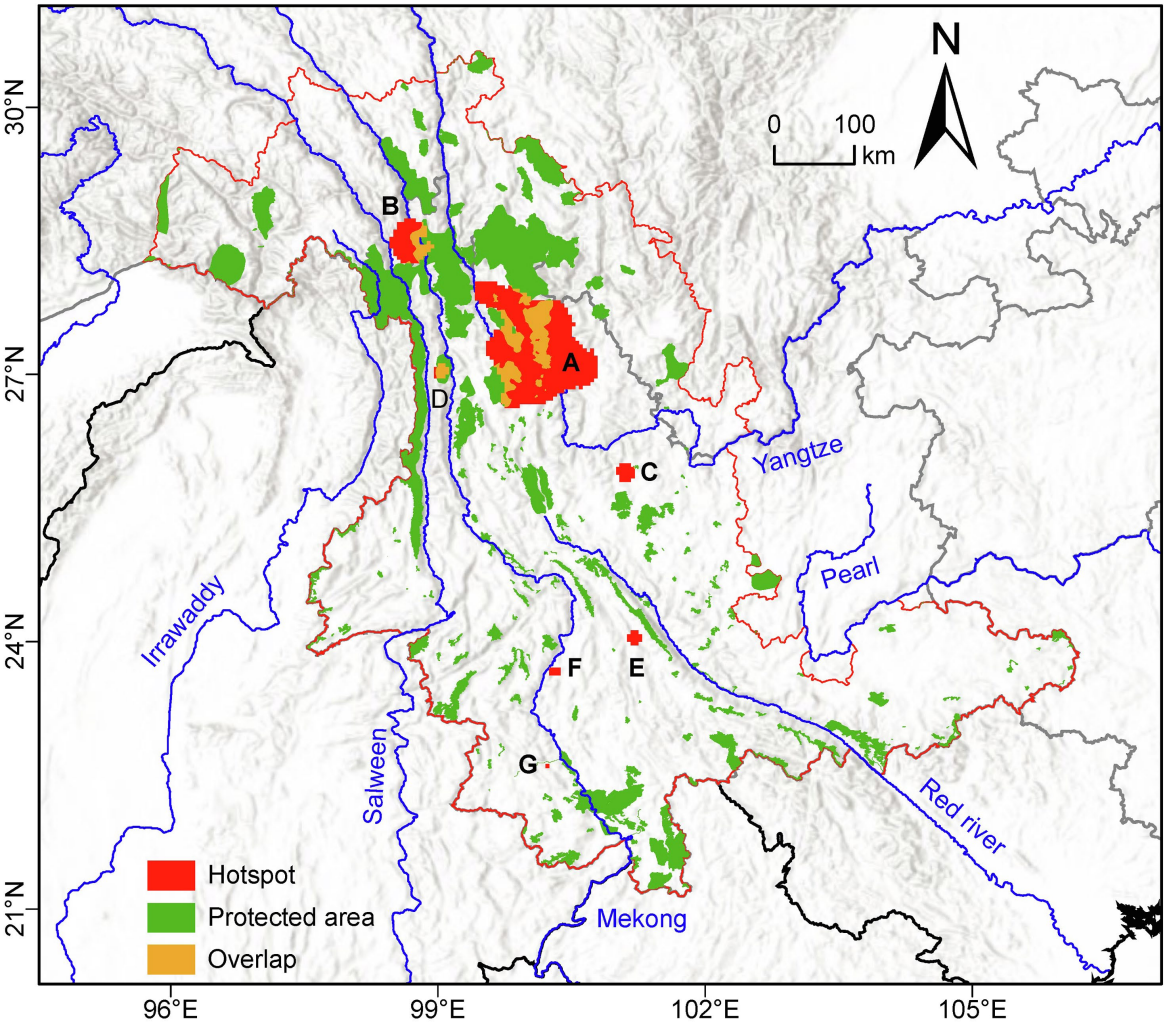
Location of the protected areas (green) and the seven genetic diversity “hotspots” (A-G; red) identified in the current study in the Longitudinal Range Gorge Region (LRGR). Areas of overlap between protected areas and “hotspots” are shown in yellow.

Based on the set threshold of 1.5 standard deviations (which corresponded to an *H*_D_ value of 0.38), we identified seven genetic diversity “hotspots” (labeled A-G in descending order of size) having a total area of 15,686.81 km^2^ (Figure 4). Of these, the largest (A) was located in the Yulong Mountain region and the second largest (B) near Meili Mountains. The remaining five were much smaller, and of these, hotspot D was located in Nushan Mountain, whereas the other 4 were distributed at lower latitudes in the southern LRGR (Figure 4). We found that about 74.81% of the genetic diversity “hotspots” land area was located outside of the established protected areas (Figure 4).

### Regression Analyses

**Figure 5:**
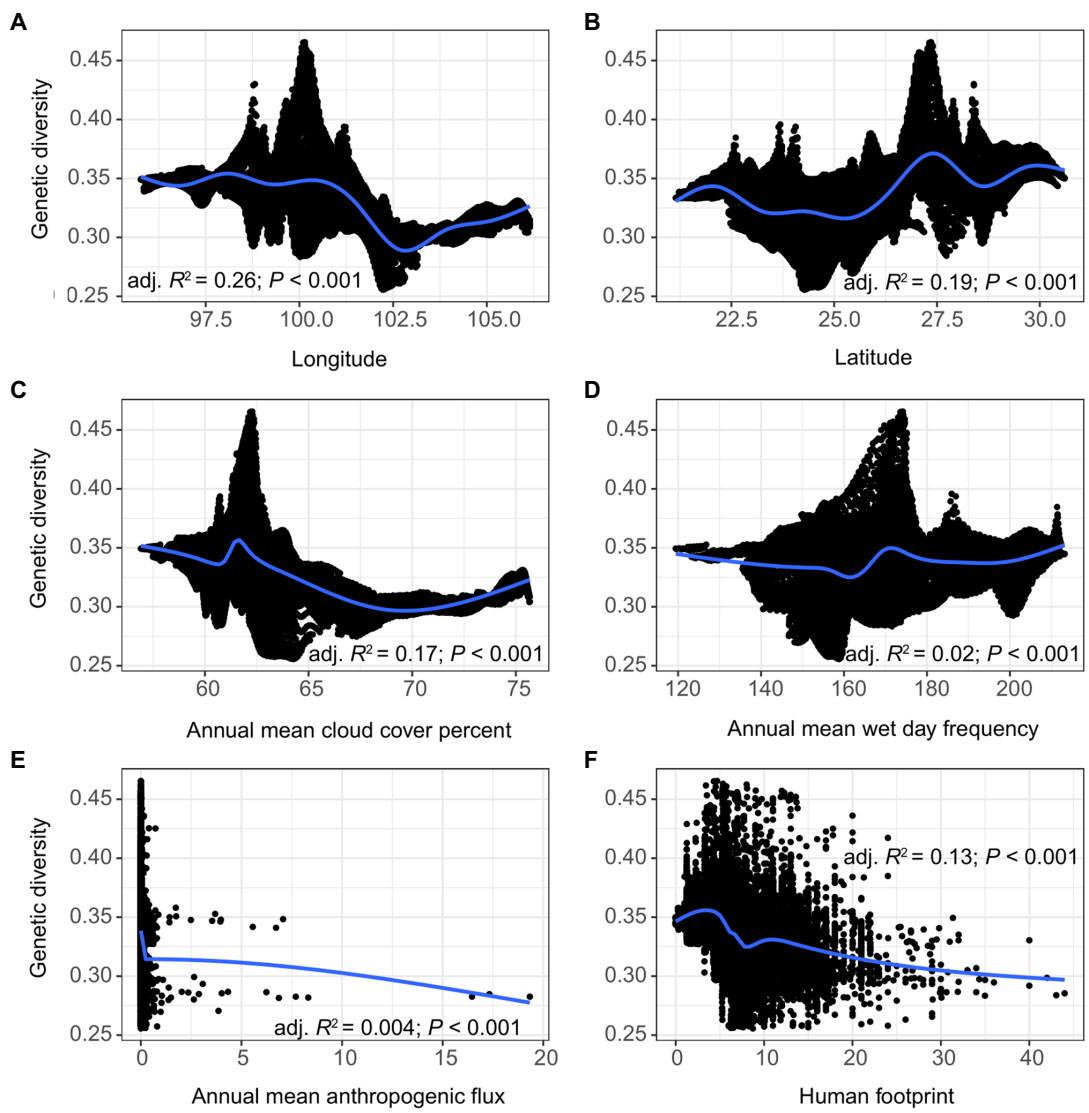
The response of genetic diversity to **(A)** Longitude, **(B)** Latitude, **(C)** Annual mean cloud cover percent, **(D)** Annual mean wet day frequency, **(E)** Annual mean anthropogenic flux, and **(F)** Human footprint index. The black shading indicates 95% confidence interval.

The model selection analysis showed that *cld*/*wet* and *lat*/*long* were the best-fitting models in the climatic and topographic categories, respectively, and these were therefore combined with the two anthropogenic variables *hfp* and *ahf* for the RDA and variation partitioning. The selected variables showed weakly negative (*long*, *cld*, *ahf*, and *hfp*) and weakly positive (*lat* and *wet*) correlation with genetic diversity, though the relationships were significant in all cases (*p* < 0.001; Figure 5). Despite these general trends, we noted slight deviations in genetic diversity along the scales of some explanatory variables. For instance, although genetic diversity generally increased with increasing *lat* (R_adj._^2^ = 0.19), there was a marked decrease in genetic diversity at ~22.5–25° and at 28.5°. A similar trend was observed for *cld* and *hfp*, in which genetic diversity increased slightly before decreasing.

**Figure 6:**
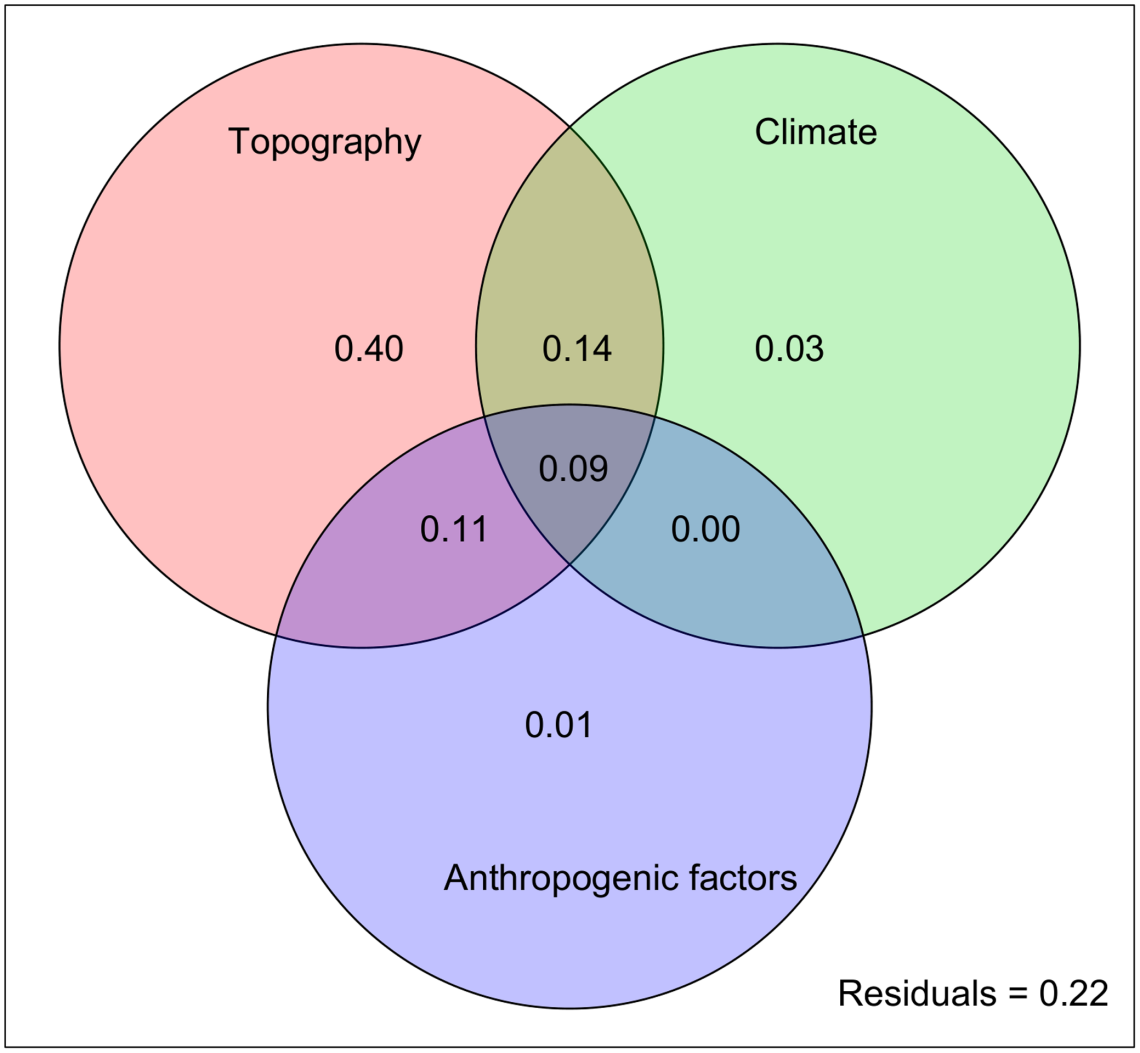
RDA partitioning results showing the relative contributions of climatic, topographic, and anthropogenic factors (and their combinations) as predictors of genetic diversity in the Longitudinal Range Gorge Region (LRGR).

In the subsequent RDA, the three categories of variables explained 78% of the total genetic variation, with topography alone accounting for the highest proportion (40%) of the variation. Climatic and anthropogenic factors explained 3 and 1% of the observed genetic variation, respectively (Figure 6). Notably, the variable combinations of climate/topography and topography/anthropogenic effects explained 14 and 11% of the total genetic variation, respectively.

## Discussion

### Patterns of Plastid Genetic Diversity and Divergence

We mapped patterns of plastid genetic diversity and divergence in the LRGR and determined the relative contributions of climatic, topographical, and anthropogenic factors in shaping the observed diversity patterns. Our results indicated that, within the LRGR, the Yulong Mountain region harbored the highest genetic diversity (Figures 3A, 4). The Yulong Mountain is located on the southeast edge of the Hengduan Mountains, a region that is known for its high species richness (Wu, 1987; Mittermeier et al., 2004) and glacial refugia (Liu et al., 2013; Jian et al., 2015). Indeed, the general distribution of genetic variation in the LRGR was reflective of the congruence between species and genetic diversity patterns. In our study, most populations were concentrated in the vicinity of the Yulong Mountain (approximately at the intersection of latitude 27°N and longitude 100°E). The high species richness of the northern LRGR is consistent with the view that orogeny (which was more intense toward the northern LRGR) was responsible for species diversification in the Himalaya/Hengduan Mountains region (Wambulwa et al., 2021). The observed congruence in species and genetic diversity patterns is a well-known phenomenon (Vellend and Geber, 2005; Vellend et al., 2014) and has been observed in both plant and animal communities (Fan et al., 2018; Hu et al., 2021).

The location of genetic diversity “hotspots” in the northern LRGR may also be explained by the fact that the Hengduan Mountains served as glacial refugia for many plant species in the region (Qiu et al., 2011 and references therein). Due to long isolation periods, populations located in refugia would be expected to harbor relatively high genetic diversity (Abbott et al., 2000; Hewitt, 2000). Since the distribution range of some of the species in our study extends northwards to the eastern Himalaya and Tibetan Plateau (e.g., Wang et al., 2011; Yang et al., 2012; Fan et al., 2013; Jian et al., 2015; Luo et al., 2018), we argue that the high genetic diversity areas observed in the current study represent the glacial refugia for some of the analyzed species (e.g., Qiu et al., 2011; Wang et al., 2011; Fan et al., 2013; Liu et al., 2013; Jian et al., 2015). Although the current study offers new insights into the distribution of genetic diversity in the region, a considerable proportion of the populations in our study lacked genetic variation, and this might have decreased the statistical power during the generation of genetic diversity landscapes and the subsequent extraction of the response variable values.

Moreover, the uneven distribution of populations in our study area might also have biased *F*_ST_ estimates. However, we found that the majority of the species did not show isolation-by-distance, suggesting the involvement of environment-driven differentiation in shaping the genetic structure of the majority of the species. The genetic divergence landscape in our study indicates the presence of gene flow barriers along a latitudinal range located approximately centrally in the LRGR, which is generally consistent with the findings of Fan et al. (2018), who mapped the genetic divergence of woody seed plants in subtropical China. Despite the insights obtained regarding the patterns of genetic diversity and divergence in the LRGR, our study may have been limited by the choice of molecular markers (cpDNA). The conserved nature of the cpDNA genome, coupled with its complex evolutionary patterns that arise from incomplete lineage sorting and interspecific introgressions, may decrease the capacity of cpDNA markers to discriminate disparate populations of a particular species.

### Determinants of Plastid Genetic Variation in the LRGR

Understanding the forces that shape the genetic structure of species across a given landscape is a fundamental goal in landscape genetics. Our analyses indicated that genetic diversity was weakly but significantly correlated with both latitude and longitude, tending to increase northwards and westwards (Figure 3A). The northern parts of the LRGR (located in the Hengduan Mountains), which harbored high levels of genetic diversity in the present study, are known for their high topographic heterogeneity. This heterogeneous landscape creates numerous and diverse within-population environmental niches that ultimately lead to strong patterns of isolation-by-environment (IBE; e.g., Liu et al., 2013). On the flip side, mountain ridges and valleys in such heterogeneous landscapes may also provide dispersal corridors among populations (Yu et al., 2017), as supported by the low levels of genetic divergence within the northern part of the LRGR in the current study (Figure 3B). The north-to-south latitudinal genetic cline is consistent with the general topology of the LRGR, i.e., the northern part of the region is characterized by extreme relief with deep river valleys while the southern part exhibits lower relief. Our results are also consistent with the idea that downwind populations are likely to harbor higher genetic diversity, as the rate of accumulation of genetic variation (caused by higher rates of net immigration) in such populations is higher than the variation that is lost due to selection or genetic drift (Kling and Ackerly, 2021). The South Asia Monsoon (Indian Summer Monsoon) blows over the LRGR in the general south to north direction; thus, a latitudinal gradient of genetic diversity would be expected in this region, although this effect may be limited to the wind-dispersed species (*Q*. *kerrii* and *Q*. *schottkyana*).

A similar trend in genetic diversity along a latitudinal gradient was recently found in subtropical evergreen oaks in southwest China (Xu et al., 2020). However, a meta-analysis of Tibetan plant species (Yu et al., 2019) did not find significant relationships between genetic diversity and either longitude or latitude, whereas Fan et al. (2018) found a positive correlation between genetic diversity and longitude in subtropical China. Although these previous studies do offer a fair axis of comparison with our results, plant communities in the two regions were subject to different historical and contemporary processes (e.g., glaciation patterns, orogeny, and climatic gradients; Qiu et al., 2011), and hence, are expected to show varying responses. Moreover, the inconsistencies could be attributed to the marked differences in spatial scale and the number of species between the two studies. In a similar scenario, a global-scale study recently found a relatively higher population genetic differentiation at lower latitudes (tropical/subtropical regions) as a result of restricted gene flow in these areas (Gamba and Muchhala, 2020). Though genetic patterns are often scale-dependent (Cushman and Landguth, 2010; Manel and Holderegger, 2013), thus rendering comparison of studies at different scales untenable, the patterns uncovered in the current study may offer useful insights into the partitioning of genetic diversity at larger spatial scales (Levy et al., 2014).

Genetic diversity had a weak but significant positive correlation with annual mean wet day frequency (*wet*) (Figure 5D). Such a link between water resources and plant genetic structure has been noted before (De Kort et al., 2021), though with precipitation being negatively related to plant genetic diversity (the correlation again weak but significant). This once again underscores the influence of spatial scale on the distribution of genetic diversity. Genetic diversity also had a weak but negative correlation with annual mean cloud cover percent (*cld*), thus reinforcing the link between genetic diversity and water. Although it is somewhat counterintuitive for *wet* and *cld* not to be correlated (they show discordant relationships with genetic diversity), the finding is not entirely unexpected, as cloud-precipitation anti-correlations have been noted particularly for low, optically thin cloud types (Jin et al., 2018). Nonetheless, our results support previous findings that climatic factors such as solar radiation, potential evaporation, and precipitation can affect the genetic structure of plant species (Bradbury et al., 2013; Kitamura et al., 2020). Although the mechanism by which such climatic factors influence genetic structure is as yet unclear, selective pressure for loci associated with photosynthesis and osmotic responses may have a role (Cruz et al., 2019). Our results hint at the importance of the interplay between energy- and water-related variables in shaping patterns of genetic diversity (e.g., Ramírez-Valiente et al., 2018; Li et al., 2019; De Kort et al., 2021), though this aspect may require a more thorough re-examination in the LRGR. As global warming is expected to cause large variations in energy and water-related factors, the evolutionary potential of most plant populations could be at risk owing to the uncertain effects of these climate changes on genetic variation. For this reason, more specific research inquiries into the role of climatic factors on plant genetic variation in the LRGR are urgently needed.

The LRGR is divided latitudinally into two climatic zones (the southern tropical monsoon and the northern subtropical monsoon climates), which might account for the relatively high proportion of genetic variation (14%) that was explained by the combination of climatic and topographic variables. Moreover, a far higher proportion (78%) of the observed genetic variation could be explained by the joint effect of climatic, topographic, and anthropogenic factors (Figure 6). This finding confirms the importance of these factors in determining the patterns of genetic structure in southwest China (e.g., Ju et al., 2018; Yang et al., 2019). Although our data show that topography is the main determinant of genetic structure in the LRGR, this relationship can be better understood in the context of climatic factors (e.g., Mosca et al., 2014; Li et al., 2019). The climatic dissimilarity and the ensuing IBE patterns in such a heterogeneous landscape (Bennie et al., 2008; Wang and Bradburd, 2014) might offer a plausible explanation for the considerably strong joint effect of climate and topography on the distribution of genetic diversity in the LRGR. The strong climate gradient in the LRGR might also explain the position of the break in genetic divergence (Figure 3B). This climatic dissimilarity, in the long run, may facilitate genetic divergence of populations along the established environmental gradients through the interaction of adaptive and non-adaptive mechanisms (Müller et al., 2017; Ramírez-Valiente et al., 2018; Galliart et al., 2019). Although longitude (*long*) and latitude (*lat*) were the best-fitting models in the topography category, other variables such as terrain roughness index (*tri*) and elevation (*elev*) might have a more practical and direct effect on genetic diversity patterns (Figure 5). We argue here that *long* and *lat* are simply proxies of these two variables (*tri* and *elev*).

Besides climate and topography, our analysis also found a weakly negative (but significant) relationship between the two anthropogenic variables and genetic diversity, suggesting the role of human activities in shaping the genetic structure of plant communities in the LRGR. However, this relationship appears to be driven partly by the huge sample size as well as other unknown outliers; thus, more anthropogenic variables may be required to clarify and validate the relationship. Though our data indicate a negative effect of human activities on the distribution of genetic diversity in the LRGR, some recent studies have alluded to the positive impact of ancient anthropogenic forces on biodiversity (e.g., Novák et al., 2019; Fajmonová et al., 2020), a trend that may also be true for genetic diversity, but more data are needed to confirm this relationship. A better understanding of the role of contemporary or recent human activities on the genetic structure of plant communities in the region would require characterization of habitats using more direct approaches. Nevertheless, the upsurge of human activities in the LRGR over recent decades is likely to exert an unprecedented pressure on natural ecosystems in the region (Liu et al., 2018), as demonstrated previously on natural reserves (including forest ecosystems) in Yunnan Province (Qiu et al., 2018).

### Implications for Biodiversity Conservation

In the wake of the current global environmental changes, it is important to preserve species adaptability and evolutionary potential, which will be achieved by considering not only species diversity but also intra-specific genetic diversity in conservation planning. Our analysis identified seven genetic diversity “hotspots” that showed an *H*_D_ value of >0.38 averaged across the study LRGR (Figure 4). As a significant proportion (~74.81%) of the identified “hotspots” land area was located outside of the protected areas, these “hotspots” should be prioritized in future conservation interventions, particularly in the context of climate change and increasing human activity. The larger “hotspots” (A, B, and D) were located in the northern part of the LRGR, where the relief is generally higher. The smaller “hotspots” (C, E, F, and G) geographically correspond to the isolated mountains in the southern part of the LRGR. However, more data from more species may be required to confirm the presence of the smaller hotspots since the southern part of the study area had relatively fewer representative species/populations. Conservation of the larger “hotspots” would require less effort since they are located closer to, and mostly overlap with, the major protected areas. However, conservation of the smaller “hotspots” may necessitate such interventions as the establishment of connections among the hotspots, as well as between the hotspots and the nearest protected areas, in order to limit gaps in the protected area network (Schoville et al., 2018). Moreover, establishment of protected areas close to cities, and corresponding to “hotspots” C, E, F, and G, would promote eco-tourism and mitigate the negative effects of urbanization on biodiversity (Conner, 2005; Puppim de Oliveira et al., 2011). To ensure sustainability and success in the conservation of these hotspot areas, it will be important for future investigations to consider population-level genetic dynamics (e.g., the effect of life history traits and the potential geophysical barriers on population-level genetic diversity) in the conservation decision-making process; conservation interventions should first recognize that the level of population genetic diversity (particularly in plants) is often a natural phenomenon that should not necessarily imply evolutionary capacity (see De Kort et al., 2021).

## Conclusion

Our results offer substantial insight into the patterns of plastid genetic structure, as well as the relative roles of climatic, topographic, and anthropogenic factors play in influencing landscape genetic patterns of plant populations in the LRGR. We identified areas that harbor high plastid genetic diversity and demonstrated the importance of topography as a determinant of genetic variation in the region. Despite the weak correlation observed between genetic diversity and anthropogenic variables, an unabated escalation of human activities in the LRGR might have devastating impacts on the future evolutionary potential of plant populations. In light of this new knowledge, relevant authorities should urgently enact policies that will address land use change, as well as high human population density, particularly within the identified genetic diversity “hotspots.” Results of the current study, however, might have been limited by two extraneous factors. First, the uneven distribution of populations in the study area (most of the species are more common in the north than in the south) is likely to have influenced the overall genetic structure to a considerable degree. Secondly, variations in life form, pollination, and dispersal modes may have biased the distribution of genetic variation, as the 15 species are not evenly distributed in the study area. We recommend that future explorations of this topic in the region should test the effect of climatic, topographic, and anthropogenic factors on genetic diversity in light of these differences in species traits. Moreover, further insights could be gained from studies that use biparentally inherited markers, which usually show higher population-level variation, and therefore allow for more meaningful inferences about the future evolutionary potential of populations within the region. Future research in the region may also benefit from genomic data, which provide more accurate estimations of genetic parameters, thus allow for more targeted measures during conservation planning.

## Data Availability Statement

The original contributions presented in the study are included in the article/Supplementary Material, further inquiries can be directed to the corresponding authors.

## Author Contributions

Z-YW, HW, L-MG, D-ZL, and JL designed the research and acquired funding. MW, JL, Y-HL, and G-FZ carried out literature search and data analysis. MW, RM, FW, and JL wrote the first draft of the manuscript. All authors contributed to interpretation of the results and editing of the manuscript.

## Funding

This study was supported by the Key Research Program of Frontier Sciences, CAS (ZDBS-LY-7001), the Strategic Priority Research Program of Chinese Academy of Sciences (XDB31010000), the National Natural Science Foundation of China (41971071, 32170389, and 31770367), and the Top-notch Young Talents Project of Yunnan Provincial “Ten Thousand Talents Program” (YNWR-QNBJ-2018-146), the CAS ‘Light of West China’ Program, and the International Partnership Program of the Chinese Academy of Sciences (151853KYSB20190027). Z-YW was supported by CAS’ Youth Innovation Promotion Association (2019385). RM thanks the CAS President’s International Fellowship Initiative (2022VBA0004). MW was supported by the Postdoctoral International Exchange Program of the Office of China Postdoctoral Council, and the Postdoctoral Targeted Funding and Postdoctoral Research Fund of Yunnan Province.

## Conflict of Interest

The authors declare that the research was conducted in the absence of any commercial or financial relationships that could be construed as a potential conflict of interest.

## Publisher’s Note

All claims expressed in this article are solely those of the authors and do not necessarily represent those of their affiliated organizations, or those of the publisher, the editors and the reviewers. Any product that may be evaluated in this article, or claim that may be made by its manufacturer, is not guaranteed or endorsed by the publisher.

## Footnotes

^1^ https://scholar.google.com/

^2^ https://www.esri.com

^3^ https://www.ncbi.nlm.nih.gov/genbank/

^4^ https://www.geneious.com

^5^ https://www.protectedplanet.net/

## References

[ref1] Abbott R. J. Smith L. C. Milne R. I. Crawford R. M. M. Wolff K. Balfour J. (2000). Molecular analysis of plant migration and refugia in the Arctic. Science 289, 1343–1346. doi: 10.1126/science.289.5483.1343, PMID: 10958779

[ref2] Badgley C. Smiley T. M. Terry R. Davis E. B. DeSantis L. R. Fox D. L.. (2017). Biodiversity and topographic complexity: modern and geohistorical perspectives. Trends Ecol. Evol. 32, 211–226. doi: 10.1016/j.tree.2016.12.010, PMID: 28196688 PMC5895180

[ref3] Bennie J. Huntley B. Wiltshire A. Hill M. O. Baxter R. (2008). Slope, aspect and climate: spatially explicit and implicit models of topographic microclimate in chalk grassland. Ecol. Model. 216, 47–59. doi: 10.1016/j.ecolmodel.2008.04.010

[ref4] Blanco-Pastor J. L. Fernández-Mazuecos M. Coello A. J. Pastor J. Vargas P. Zhan A. (2019). Topography explains the distribution of genetic diversity in one of the most fragile European hotspots. Divers. Distrib. 25, 74–89. doi: 10.1111/ddi.12836

[ref5] Borcard D. (1992). Partialling out the spatial component of ecological variation. Ecology 73, 1045–1055. doi: 10.2307/1940179

[ref6] Bradbury D. Smithson A. Krauss S. L. (2013). Signatures of diversifying selection at EST-SSR loci and association with climate in natural *eucalyptus* populations. Mol. Ecol. 22, 5112–5129. doi: 10.1111/mec.12463, PMID: 24118117

[ref7] Carnaval A. C. Hickerson M. J. Haddad C. F. B. Rodrigues M. T. Moritz C. (2009). Stability predicts genetic diversity in the Brazilian Atlantic forest hotspot. Science 323, 785–789. doi: 10.1126/science.1166955, PMID: 19197066

[ref9] Chen C. Y. Liang B. K. Chung J. D. Chang C. T. Hsieh Y. C. Lin T. C.. (2014). Demography of the upward-shifting temperate woody species of the *rhododendron pseudochrysanthum* complex and ecologically relevant adaptive divergence in its trailing edge populations. Tree Genet. Genomes 10, 111–126. doi: 10.1007/s11295-013-0669-x

[ref10] Cheng Q. Gao L. Zhong F. Zuo X. Ma M. (2020). Spatiotemporal variations of drought in the Yunnan-Guizhou plateau, Southwest China, during 1960–2013 and their association with large-scale circulations and historical records. Ecol. Indic. 112:106041. doi: 10.1016/j.ecolind.2019.106041

[ref11] Conner N. (2005). “Some benefits of protected areas for urban communities: a view from Sydney, Australia,” in The Urban Imperative. ed. Trzyna T. (Sacramento: California Institute of Public Affairs).

[ref12] Cruz M. V. Mori G. M. Signori-Müller C. da Silva C. C. Oh D. H. Dassanayake M.. (2019). Local adaptation of a dominant coastal tree to freshwater availability and solar radiation suggested by genomic and ecophysiological approaches. Sci. Rep. 9:19936. doi: 10.1038/s41598-019-56469-w, PMID: 31882752 PMC6934818

[ref13] Cushman S. A. Landguth E. L. (2010). Scale dependent inference in landscape genetics. Landsc. Ecol. 25, 967–979. doi: 10.1007/s10980-010-9467-0 20618896

[ref14] De Kort H. Prunier J. G. Ducatez S. Honnay O. Baguette M. Stevens V. M.. (2021). Life history, climate and biogeography interactively affect worldwide genetic diversity of plant and animal populations. Nat. Commun. 12:516. doi: 10.1038/s41467-021-20958-2, PMID: 33483517 PMC7822833

[ref15] De Villemereuil P. Mouterde M. Gaggiotti O. E. Till-Bottraud I. (2018). Patterns of phenotypic plasticity and local adaptation in the wide elevation range of the alpine plant *Arabis alpina*. J. Ecol. 106, 1952–1971. doi: 10.1111/1365-2745.12955

[ref16] Di Marco M. Harwood T. D. Hoskins A. J. Ware C. Hill S. L. Ferrier S. (2019). Projecting impacts of global climate and land-use scenarios on plant biodiversity using compositional-turnover modelling. Glob. Change Biol. 25, 2763–2778. doi: 10.1111/gcb.14663, PMID: 31009149

[ref17] Duan X. Gu Z. Li Y. Xu H. (2016). The spatiotemporal patterns of rainfall erosivity in Yunnan Province, Southwest China: an analysis of empirical orthogonal functions. Glob. Planet. Change 144, 82–93. doi: 10.1016/j.gloplacha.2016.07.011

[ref18] Ellegren H. Galtier N. (2016). Determinants of genetic diversity. Nat. Rev. 17, 422–433. doi: 10.1038/nrg.2016.58 27265362

[ref19] Escudero A. Iriondo J. M. Torres M. E. (2003). Spatial analysis of genetic diversity as a tool for plant conservation. Biol. Conserv. 113, 351–365. doi: 10.1016/S0006-3207(03)00122-8

[ref20] Excoffier L. Lischer H. (2010). Arlequin suite ver 3.5: a new series of programs to perform population genetics analyses under Linux and windows. Mol. Ecol. Resour. 10, 564–567. doi: 10.1111/j.1755-0998.2010.02847.x, PMID: 21565059

[ref21] Fajmonová Z. Hájková P. Hájek M. (2020). Soil moisture and a legacy of prehistoric human activities have contributed to the extraordinary plant species diversity of grasslands in the White Carpathians. Preslia 92, 35–56. doi: 10.23855/preslia.2020.035

[ref22] Fan Z. X. Brauning A. Thomas A. Li J. B. Cao K. F. (2011). Spatial and temporal temperature trends on the Yunnan plateau (Southwest China) during 1961–2004. Int. J. Climatol. 31, 2078–2090. doi: 10.1002/joc.2214

[ref23] Fan D. Huang J. Hu H. Sun Z. Cheng S. Kou Y.. (2018). Evolutionary hotspots of seed plants in subtropical China: a comparison with species diversity hotspots of woody seed plants. Front. Genet. 9:333. doi: 10.3389/fgene.2018.00333, PMID: 30177954 PMC6109751

[ref24] Fan D. Sun Z. Li B. Kou Y. Hodel R. G. J. Jin Z.. (2017). Dispersal corridors for plant species in the Poyang Lake Basin of Southeast China identified by integration of phylogeographic and geospatial data. Ecol. Evol. 7, 5140–5148. doi: 10.1002/ece3.2999, PMID: 28770054 PMC5528243

[ref25] Fan D. M. Yue J. P. Nie Z. L. Li Z. M. Comes H. P. Sun H. (2013). Phylogeography of *Sophora davidii* (Leguminosae) across the 'Tanaka-Kaiyong Line', an important phytogeographic boundary in Southwest China. Mol. Ecol. 22, 4270–4288. doi: 10.1111/mec.12388, PMID: 23927411

[ref26] FAO (2018). World Food and Agriculture Statistical Pocketbook 2018. Rome: FAO.

[ref27] Farr T. G. Rosen P. A. Caro E. Crippen R. Duren R. Hensley S.. (2007). The shuttle radar topography mission. Rev. Geophys. 45:RG2004. doi: 10.1029/2005RG000183

[ref28] Feeley K. J. Silman M. R. (2010). Land-use and climate change effects on population size and extinction risk of Andean plants. Glob. Chang. Biol. 16, 3215–3222. doi: 10.1111/j.1365-2486.2010.02197.x

[ref29] Fenderson L. E. Kovach A. I. Llamas B. (2019). Spatiotemporal landscape genetics: investigating ecology and evolution through space and time. Mol. Ecol. 29, 218–246. doi: 10.1111/mec.15315, PMID: 31758601

[ref30] Frankham R. Ballou J. Briscoe D. (2010). Introduction to Conservation Genetics. UK: Cambridge University Press.

[ref31] Galliart M. Bello N. Knapp M. Poland J. Amand P. Baer S.. (2019). Local adaptation, genetic divergence, and experimental selection in a foundation grass across the US Great Plains’ climate gradient. Glob. Change Biol. 25, 850–868. doi: 10.1111/gcb.14534, PMID: 30468548

[ref32] Gamba D. Muchhala N. (2020). Global patterns of population genetic differentiation in seed plants. Mol. Ecol. 29, 3413–3428. doi: 10.1111/mec.15575, PMID: 32743850

[ref33] Gao L. Moller M. Zhang X. Hollingsworth M. Liu J. Mill R.. (2007). High variation and strong phylogeographic pattern among cpDNA haplotypes in *Taxus wallichiana* (Taxaceae) in China and North Vietnam. Mol. Ecol. 16, 4684–4698. doi: 10.1111/j.1365-294X.2007.03537.x, PMID: 17908214

[ref34] Hewitt G. (2000). The genetic legacy of the quaternary ice ages. Nature 405, 907–913. doi: 10.1038/35016000, PMID: 10879524

[ref35] HuY.FanH.ChenY.ChangJ.ZhanX.WuH.. (2021). Spatial patterns and conservation of genetic and phylogenetic diversity of wildlife in China. Sci. Adv. 7:eabd5725. doi: 10.1126/sciadv.abd5725, PMID: 33523945

[ref36] Huang D. Q. Li Q. Q. Zhou C. J. Zhou S. D. He X. J. (2014). Intraspecific differentiation of *Allium wallichii* (Amaryllidaceae) inferred from chloroplast DNA and internal transcribed spacer fragments. J. Syst. Evol. 52, 341–354. doi: 10.1111/jse.12050

[ref37] Jian H. Li S. Guo J. Li S. Wang Q. Yan H.. (2018). High genetic diversity and differentiation of an extremely narrowly distributed and critically endangered decaploid rose (*Rosa praelucens*): implications for its conservation. Conserv. Genet. 19, 761–776. doi: 10.1007/s10592-018-1052-0

[ref38] Jian H. Y. Tang K. X. Sun H. (2015). Phylogeography of *Rosa soulieana* (Rosaceae) in the Hengduan Mountains: Refugia and ‘melting’ pots in the quaternary climate oscillations. Plant Syst. Evol. 301, 1819–1830. doi: 10.1007/s00606-015-1195-0

[ref39] Jiang X. L. An M. Zheng S. S. Deng M. Su Z. H. (2018). Geographical isolation and environmental heterogeneity contribute to the spatial genetic patterns of *Quercus kerrii* (Fagaceae). Heredity 120, 219–233. doi: 10.1038/s41437-017-0012-7, PMID: 29279604 PMC5836588

[ref40] Jiang X. L. Deng M. Li Y. (2016). Evolutionary history of subtropical evergreen broad-leaved forest in Yunnan plateau and adjacent areas: an insight from *Quercus schottkyana* (Fagaceae). Tree Genet. Genomes 12:104. doi: 10.1007/s11295-016-1063-2

[ref41] Jin D. Oreopoulos L. Lee D. Cho N. Tan J. (2018). Contrasting the co-variability of daytime cloud and precipitation over tropical land and ocean. Atmos. Chem. Phys. 18, 3065–3082. doi: 10.5194/acp-18-3065-2018, PMID: 32661461 PMC7356930

[ref42] Ju M. M. Fu Y. Zhao G. F. He C. Z. Li Z. H. Tian B. (2018). Effects of the Tanaka line on the genetic structure of *Bombax ceiba* (Malvaceae) in dry-hot valley areas of Southwest China. Ecol. Evol. 8, 3599–3608. doi: 10.1002/ece3.3888, PMID: 29686841 PMC5901178

[ref43] Katoh K. Standley D. M. (2013). MAFFT multiple sequence alignment software version 7: improvements in performance and usability. Mol. Biol. Evol. 30, 772–780. doi: 10.1093/molbev/mst010, PMID: 23329690 PMC3603318

[ref44] Kitamura K. Uchiyama K. Ueno S. Ishizuka W. Tsuyama I. Goto S. (2020). Geographical gradients of genetic diversity and differentiation among the southernmost marginal populations of *Abies sachalinensis* revealed by EST-SSR polymorphism. Forests 11:233. doi: 10.3390/f11020233

[ref45] Kling M. M. Ackerly D. D. (2021). Global wind patterns shape genetic differentiation, asymmetric gene flow, and genetic diversity in trees. Proc. Natl. Acad. Sci. U. S. A. 118:e2017317118. doi: 10.1073/pnas.2017317118, PMID: 33875589 PMC8092467

[ref46] Lenoir J. Gégout J. Marquet P. de Ruffray P. Brisse H. (2008). A significant upward shift in plant species optimum elevation during the 20th century. Science 320, 1768–1771. doi: 10.1126/science.1156831, PMID: 18583610

[ref47] Levy O. Ball B. A. Punyasena S. W. Xiao J. Bond-Lamberty B. Cheruvelil K. S.. (2014). Approaches to advance scientific understanding of macrosystems ecology. Front. Ecol. Environ. 12:130019. doi: 10.1890/130019

[ref48] Li G. D. Yue L. L. Sun H. Qian Z. G. (2012). Phylogeography of *Cyananthus delavayi* (Campanulaceae) in Hengduan Mountains inferred from variation in nuclear and chloroplast DNA sequences. J. Syst. Evol. 50, 305–315. doi: 10.1111/j.1759-6831.2012.00200.x

[ref49] Li Y. Zhai S. N. Qiu Y. X. Guo Y. P. Ge X. J. Comes H. P. (2011). Glacial survival east and west of the 'Mekong-Salween Divide' in the Himalaya-Hengduan Mountains region as revealed by AFLPs and cpDNA sequence variation in *Sinopodophyllum hexandrum* (Berberidaceae). Mol. Phylogenet. Evol. 59, 412–424. doi: 10.1016/j.ympev.2011.01.009, PMID: 21296173

[ref50] Li Y. Zhang X. Fang Y. (2019). Landscape features and climatic forces shape the genetic structure and evolutionary history of an oak species (*Quercus chenii*) in East China. Front. Plant Sci. 10:1060. doi: 10.3389/fpls.2019.01060, PMID: 31552065 PMC6734190

[ref51] Liang J. Liu Y. Ying L. Li P. Xu Y. Shen Z. (2014). Road impacts on spatial patterns of land use and landscape fragmentation in three parallel Rivers region, Yunnan Province. China. Chin. Geogr. Sci. 24, 15–27. doi: 10.1007/s11769-014-0652-y

[ref52] Liaw A. Wiener M. (2002). Classification and regression by randomForest. R News 2, 18–22.

[ref53] Librado P. Rozas J. (2009). DnaSP v5: a software for comprehensive analysis of DNA polymorphism data. Bioinformatics 25, 1451–1452. doi: 10.1093/bioinformatics/btp187, PMID: 19346325

[ref55] Liu J. Cui B. S. Yang Z. F. Dong S. K. Yao W. K. (2006a). Effects caused by highway construction on plant biomass of roadsides in longitudinal range-gorge region. Acta Ecol. Sin. 26, 83–90. (in Chinese).

[ref200] Liu J. Dudley N. Alexander S. (2017). Agriculture and biodiversity: a review. Biodiversity 18, 45–49. doi: 10.1080/14888386.2017.1351892

[ref300] Liu J. Milne R. I. Cadotte M. W. Wu Z. Y. Provan J. Zhu G. F.. (2018). Protect Third Pole’s fragile ecosystem. Science 362:1368. doi: 10.1126/science.aaw0443, PMID: 30573620

[ref56] Liu J. Möller M. Provan J. Gao L.-M. Poudel R. C. Li D.-Z. (2013). Geological and ecological factors drive cryptic speciation of yews in a biodiversity hotspot. New Phytol. 199, 1093–1108. doi: 10.1111/nph.12336 23718262

[ref57] Liu S. L. Wen M. X. Cui B. S. Dong S. K. (2006b). Effects of road networks on regional ecosystems in southwest mountain area: a case study in Jinhong of longitudinal range-gorge region. Acta Ecol. Sin. 26, 3018–3024. (in Chinese).

[ref58] Luo D. Xu B. Li Z. Sun H. (2017). The ‘Ward line–Mekong–Salween divide’ is an important floristic boundary between the eastern Himalaya and Hengduan Mountains: evidence from the phylogeographical structure of subnival herbs *Marmoritis complanatum* (Lamiaceae). Bot. J. Linn. Soc. 185, 482–496. doi: 10.1093/botlinnean/box067

[ref59] Luo D. Xu B. Rana S. K. Li Z. M. Sun H. (2018). Phylogeography of rare fern *Polystichum glaciale* endemic to the subnival zone of the Sino-Himalaya. Plant Syst. Evol. 304, 485–499. doi: 10.1007/s00606-018-1495-2

[ref60] Mairal M. Sanmartín I. Herrero A. Pokorny L. Vargas P. Aldasoro J. J.. (2017). Geographic barriers and Pleistocene climate change shaped patterns of genetic variation in the eastern Afromontane biodiversity hotspot. Sci. Rep. 7:45749. doi: 10.1038/srep45749, PMID: 28397796 PMC5387718

[ref62] Manel S. Holderegger R. (2013). Ten years of landscape genetics. Trends Ecol. Evol. 28, 614–621. doi: 10.1016/j.tree.2013.05.012 23769416

[ref63] Meng L. Chen G. Li Z. Yang Y. Wang Z. Wang L. (2015). Refugial isolation and range expansions drive the genetic structure of *Oxyria sinensis* (Polygonaceae) in the Himalaya-Hengduan Mountains. Sci. Rep. 5:10396. doi: 10.1038/srep10396, PMID: 26013161 PMC4445061

[ref64] Mittermeier R. A. Gil P. R. Hoffmann M. Pilgrim J. Brooks T. Mittermeier C. G.. (2004). Hotspots Revisited. Earth's Biologically Richest and Most Endangered Terrestrial Ecoregions. Mexico City: Cemex.

[ref65] Mosca E. Gonzalez-Martınez S. C. Neale D. B. (2014). Environmental versus geographical determinants of genetic structure in two subalpine conifers. New Phytol. 201, 180–192. doi: 10.1111/nph.12476, PMID: 24102203

[ref66] Müller C. Schulz B. Lauterbach D. Ristow M. Wissemann V. Gemeinholzer B. (2017). *Geropogon hybridus* (L.) Sch. Bip. (Asteraceae) exhibits micro-geographic genetic divergence at ecological range limits along a steep precipitation gradient. Plant Syst. Evol. 303, 91–104. doi: 10.1007/s00606-016-1354-y

[ref67] Nei M. (1973). Analysis of gene diversity in subdivided populations. Proc. Natl. Acad. Sci. U. S. A. 70, 3321–3323. doi: 10.1073/pnas.70.12.3321, PMID: 4519626 PMC427228

[ref68] Nei M. (1987). Molecular Evolutionary Genetics. New York, NY, USA: Columbia University Press.

[ref69] Novák J. Roleček J. Dresler P. Hájek M. (2019). Soil charcoal elucidates the role of humans in the development of landscape of extreme biodiversity. Land Degrad. Dev. 30, 1607–1619. doi: 10.1002/ldr.3350

[ref70] Ohsawa T. Ide Y. (2008). Global patterns of genetic variation in plant species along vertical and horizontal gradients on mountains. Global Ecol. Bogeogr. 17, 152–163. doi: 10.1111/j.1466-8238.2007.00357.x

[ref71] Oksanen J. Guillaume B. F. Kindt R. Legendre P. Minchin P. O’Hara R.. (2011). Vegan: Community Ecology Package. R Package ver. 2.0-2.

[ref001] Pan T. Wu S. He D. Dai E. Liu Y. (2012). Effects of longitudinal range-gorge terrain on the eco-geographical pattern in Southwest China. J. Geogr. Sci. 22, 825–842. doi: 10.1007/s11442-012-0967-5

[ref72] Pu Y. S. Zhang Z. Y. Pu L. N. Hui C. M. (2007). Biodiversity and its fragility in Yunnan. China. J. For. Res. 18, 39–47. doi: 10.1007/s11676-007-0008-x

[ref73] Puppim de Oliveira J. A. Balaban O. Doll C. N. H. Moreno-Peñaranda R. Gasparatos A. Iossifova D.. (2011). Cities and biodiversity: perspectives and governance challenges for implementing the convention on biological diversity (CBD) at the city level. Biol. Conserv. 144, 1302–1313. doi: 10.1016/j.biocon.2010.12.007

[ref74] Qiu Y.-X. Fu C.-X. Comes H. P. (2011). Plant molecular phylogeography in China and adjacent regions: tracing the genetic imprints of quaternary climate and environmental change in the world’s most diverse temperate flora. Mol. Phylogenet. Evol. 59, 225–244. doi: 10.1016/j.ympev.2011.01.012, PMID: 21292014

[ref75] Qiu C. Hu J. Yang F. Liu F. Li X. (2018). Human pressures on natural reserves in Yunnan Province and management implications. Sci. Rep. 8:3260. doi: 10.1038/s41598-018-21654-w, PMID: 29459749 PMC5818542

[ref76] Ramírez-Valiente J. Deacon N. Etterson J. Center A. Sparks J. Sparks K.. (2018). Natural selection and neutral evolutionary processes contribute to genetic divergence in leaf traits across a precipitation gradient in the tropical oak *Quercus oleoides*. Mol. Ecol. 27, 2176–2192. doi: 10.1111/mec.14566, PMID: 29577469

[ref77] Raubeson L. Jansen R. Henry R. J. (2005). “Diversity and Evolution of Plants-Genotypic and Phenotypic Variation in Higher Plants,” in Chloroplast Genomes of Plants. ed. Henry R. J. (Wallingford: CABI Publishing).

[ref78] Schoville S. D. Dalongeville A. Viennois G. Gugerli F. Taberlet P. Lequette B.. (2018). Preserving genetic connectivity in the European Alps protected area network. Biol. Conserv. 218, 99–109. doi: 10.1016/j.biocon.2017.12.017

[ref79] Shi P. Wu M. Qu S. Jiang P. Qiao X. Chen X.. (2015). Spatial distribution and temporal trends in precipitation concentration indices for the Southwest China. Water Resour. Manag. 29, 3941–3955. doi: 10.1007/s11269-015-1038-3

[ref80] Shrestha N. Xu X. Meng J. Wang Z. (2021). Vulnerabilities of protected lands in the face of climate and human footprint changes. Nat. Commun. 12:1632. doi: 10.1038/s41467-021-21914-w, PMID: 33712613 PMC7955075

[ref81] Tajima F. (1983). Evolutionary relationship of DNA sequences in finite populations. Genetics 105, 437–460. doi: 10.1093/genetics/105.2.437, PMID: 6628982 PMC1202167

[ref82] Tamura K. Nei M. (1993). Estimation of the number of nucleotide substitutions in the control region of mitochondrial DNA in humans and chimpanzees. Mol. Biol. Evol. 10, 512–526. doi: 10.1093/oxfordjournals.molbev.a040023, PMID: 8336541

[ref83] Tang C. Matsui T. Ohashi H. Dong Y. F. Momohara A. Herrando-Moraira S.. (2018). Identifying long-term stable refugia for relict plant species in East Asia. Nat. Commun. 9:4488. doi: 10.1038/s41467-018-06837-3, PMID: 30367062 PMC6203703

[ref85] Thuiller W. Lavorel S. Araujo M. B. Sykes M. T. Prentice I. C. (2005). Climate change threats to plant diversity in Europe. Proc. Natl. Acad. Sci. U. S. A. 102, 8245–8250. doi: 10.1073/pnas.0409902102, PMID: 15919825 PMC1140480

[ref86] Tian S. Kou Y. Zhang Z. Yuan L. Li D. López-Pujol J.. (2018). Phylogeography of *Eomecon chionantha* in subtropical China: the dual roles of the Nanling Mountains as a glacial refugium and a dispersal corridor. BMC Evol. Biol. 18:20. doi: 10.1186/s12862-017-1093-x, PMID: 29426277 PMC5807764

[ref87] UNESCO (2010). Three Parallel Rivers of Yunnan Protected Areas. Available at: https://whc.unesco.org/en/list/1083 (Accessed August 13, 2021).

[ref88] Vandergast A. Bohonak A. Hathaway S. Boys J. Fisher R. (2008). Are hotspots of evolutionary potential adequately protected in southern California? Biol. Conserv. 141, 1648–1664. doi: 10.1016/j.biocon.2008.04.009

[ref89] Vandergast A. Perry W. Lugo R. Hathaway S. (2011). Genetic landscapes GIS toolbox: tools to map patterns of genetic divergence and diversity. Mol. Ecol. Resour. 11, 158–161. doi: 10.1111/j.1755-0998.2010.02904.x, PMID: 21429115

[ref90] Vellend M. Geber M. A. (2005). Connections between species diversity and genetic diversity. Ecol. Lett. 8, 767–781. doi: 10.1111/j.1461-0248.2005.00775.x

[ref91] Vellend M. Lajoie G. Bourret A. Murria C. Kembel S. W. Garant D. (2014). Drawing ecological inferences from coincident patterns of population- and community-level biodiversity. Mol. Ecol. 23, 2890–2901. doi: 10.1111/mec.12756, PMID: 24750409

[ref92] Wambulwa M. C. Milne R. Wu Z. Y. Spicer R. A. Provan J. Luo Y. H.. (2021). Spatiotemporal maintenance of flora in the Himalaya biodiversity hotspot: current knowledge and future perspectives. Ecol. Evol. 11, 10794–10812. doi: 10.1002/ece3.7906, PMID: 34429882 PMC8366862

[ref93] Wang I. Bradburd G. (2014). Isolation by environment. Mol. Ecol. 23, 5649–5662. doi: 10.1111/mec.12938 25256562

[ref94] Wang J. F. Pan Y. Z. Gong X. Chiang Y. C. Kuroda C. (2011). Chloroplast DNA variation and phylogeography of *Ligularia tongolensis* (Asteraceae), a species endemic to the Hengduan Mountains region of China. J. Syst. Evol. 49, 108–119. doi: 10.1111/j.1759-6831.2011.00117.x

[ref95] Wood S. N. (2017). Generalized Additive Models: An Introduction with R. Boca Raton, Forida: Chapman and Hall/CRC.

[ref96] Wu Z. (1987). Vegetation of Yunnan. Beijing: Science Press.

[ref98] Xu J. Song Y. G. Deng M. Jiang X. L. Zheng S. S. Li Y. (2020). Seed germination schedule and environmental context shaped the population genetic structure of subtropical evergreen oaks on the Yun-Gui plateau, Southwest China. Heredity 124, 499–513. doi: 10.1038/s41437-019-0283-2, PMID: 31772317 PMC7028733

[ref99] Xu W. Xiao Y. Zhang J. Yang W. Zhang L. Hull V.. (2017). Strengthening protected areas for biodiversity and ecosystem services in China. Proc. Natl. Acad. Sci. U. S. A. 114, 1601–1606. doi: 10.1073/pnas.1620503114, PMID: 28137858 PMC5321011

[ref100] Yang Z. Y. Yi T. S. Pan Y. Z. Gong X. (2012). Phylogeography of an alpine plant *Ligularia vellerea* (Asteraceae) in the Hengduan Mountains. J. Syst. Evol. 50, 316–324. doi: 10.1111/j.1759-6831.2012.00199.x

[ref101] Yang A. Zhong Y. Liu S. Liu L. Liu T. Li L.. (2019). New insight into the phylogeographic pattern of *Liriodendron chinense* (Magnoliaceae) revealed by chloroplast DNA: east–west lineage split and genetic mixture within western subtropical China. PeerJ 7:e6355. doi: 10.7717/peerj.6355, PMID: 30723627 PMC6361005

[ref400] Yong D. L. Choi C. Y. Gibson L. (2020). Transboundary frontiers: an emerging priority for biodiversity conservation. Trend Ecol. Evol. 35, 679–690. doi: 10.1016/j.tree.2020.03.004 32668213

[ref500] You Z. Hu J. Wei Q. Li C. Deng X. Jiang Z. (2018). Pitfall of big databases. Proc. Natl. Acad. Sci. U. S. A. 115:E9026. doi: 10.1073/pnas.1813323115 30217897 PMC6166826

[ref102] Yu H. Favre A. Sui X. Chen Z. Qi W. Xie G.. (2019). Mapping the genetic patterns of plants in the region of the Qinghai-Tibet plateau: implications for conservation strategies. Divers. Distrib. 25, 310–324. doi: 10.1111/ddi.12847

[ref103] Yu H. Zhang Y. Wang Z. Liu L. Chen Z. Qi W. (2017). Diverse range dynamics and dispersal routes of plants on the Tibetan plateau during the late quaternary. PLoS One 12:e0177101. doi: 10.1371/journal.pone.0177101, PMID: 28475607 PMC5419580

[ref104] Yue L. L. Chen G. Sun W. B. Sun H. (2012). Phylogeography of *Buddleja crispa* (Buddlejaceae) and its correlation with drainage system evolution in southwestern China. Am. J. Bot. 99, 1726–1735. doi: 10.3732/ajb.1100506, PMID: 23024123

[ref105] Zhao Y. J. Gong X. (2015). Genetic divergence and phylogeographic history of two closely related species (*Leucomeris decora* and *Nouelia insignis*) across the 'Tanaka Line' in Southwest China. BMC Evol. Biol. 15:134. doi: 10.1186/s12862-015-0374-5, PMID: 26153437 PMC4495643

[ref106] Zheng X. Kang W. Zhao T. Luo Y. Duan C. Chen J. (2008). Long-term trends in sunshine duration over Yunnan-Guizhou plateau in Southwest China for 1961–2005. Geophys. Res. Lett. 35:L15707. doi: 10.1029/2008GL034482

